# Subcutaneous immunotherapy in a patient taking ofatumumab for multiple sclerosis and upadacitinib for atopic dermatitis

**DOI:** 10.1016/j.jacig.2025.100411

**Published:** 2025-01-17

**Authors:** Twan Sia, Leeon Bacchus, Stanley Liu, John Leung

**Affiliations:** a Boston Specialists, Boston, Mass; b Stanford University School of Medicine, Stanford, Calif

**Keywords:** SCIT, allergic rhinitis, MS, ofatumumab, upadacitinib, CD20, JAK inhibitor, B cell

## Abstract

Allergen-specific immunotherapy has not been well-studied in the setting of increasingly common immune system-targeting medications. Subcutaneous immunotherapy may not be contraindicated in patients taking anti-CD20 mAbs antibodies and/or Janus kinase inhibitors.

Allergen-specific subcutaneous immunotherapy (SCIT) is the best-established treatment for modulating immune responses in allergic rhinitis, with strong evidence demonstrating its safety and efficacy.^1^ The current mechanistic understanding of SCIT suggests changes in memory-type allergen-specific T- and B-cell responses, including an upregulation of allergen-specific IL-10–producing regulatory T cells and regulatory B cells, coupled with decreases in allergen-specific T_H_2 cells.^2^ However, our understanding of the molecular pathways underlying SCIT is likely incomplete. With the advent of new medication classes targeting immunologic pathways, it is important to understand how these medications affect the safety and efficacy of SCIT when administered concurrently.

Janus kinases (JAKs) facilitate cytokine receptor signal transduction. Several nonspecific and specific JAK inhibitors have been approved for several conditions, including myeloproliferative neoplasms, rheumatoid arthritis, inflammatory bowel disease, and atopic dermatitis.^3^ Separately, CD20 is a B-cell surface marker implicated in B-cell signaling. Anti-CD20 mAbs deplete B cells in the treatment of lymphoma, multiple sclerosis (MS), and other autoimmune conditions.^4^ To date, there have not been any reports of initiation of SCIT in patients taking JAK inhibitors or anti-CD20 mAbs.

In this case report, we describe the treatment course of SCIT in a patient taking ofatumumab (an anti-CD20 mAb) for MS and upadacitinib (a selective JAK-1 inhibitor) for atopic dermatitis. Written informed consent was obtained from the patient.

We present the case of a 35-year-old female with relapse remitting MS, atopic dermatitis, and allergic rhinitis and conjunctivitis. She was diagnosed with MS at the age of 26 years by magnetic resonance imaging, with her symptoms including internuclear ophthalmoparesis, sensory symptoms, gait imbalance, limb spasticity, urinary urgency, and depression. Her baseline MS Impact Scale (MSIS-29) score was 73 (the maximum score is 145, which corresponds to most severe MS, and the minimum score is 29) (Fig 1). Administration of several medications was initiated and discontinued because of adverse effects. These medications included subcutaneous glatiramer acetate (which resulted in an immediate postinjection reaction including flushing, urticaria, and dyspnea) and subcutaneous IFN-β1a (influenza-like symptoms). She began taking subcutaneous ofatumumab, 20 mg every 4 weeks, with improvement of her symptoms (her MSIS-29 score was 34). No adverse effects were attributable to ofatumumab.

**Fig 1:**
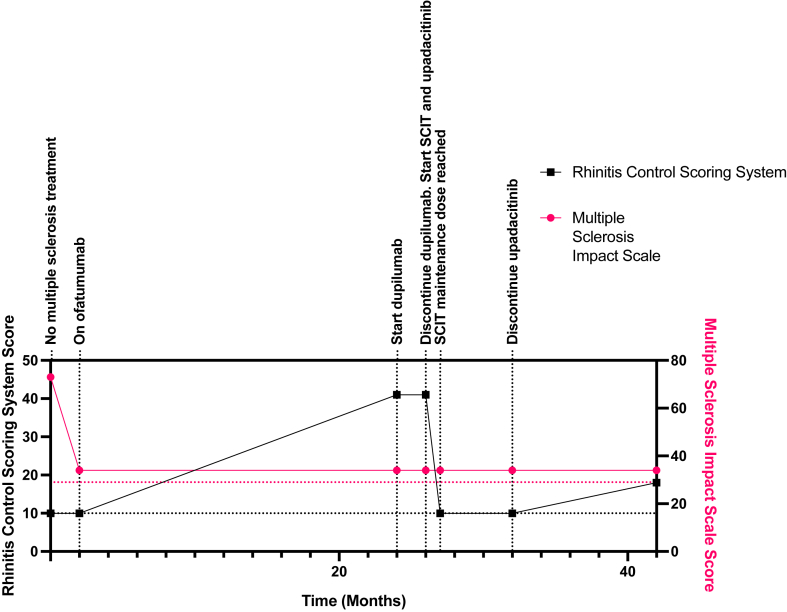
Allergic rhinitis (*black*) and MS (*magenta*) disease course in time (months). Allergic rhinitis severity is measured using the RCSS score, and MS severity is measured using Multiple Sclerosis Impact Scale (MSIS-29) score. *Black horizontal dotted line* indicates the minimum (best) RCSS score of 10, and *magenta horizontal dotted line* indicates the minimum (best) MSIS-29 score of 29.

Several months after the patient had started taking ofatumumab, she began developing atopic dermatitis along with allergic rhinitis and conjunctivitis. Her atopic dermatitis appeared on the flexor surfaces of her elbows and knees, abdomen, shoulders, and back (approximately 25% body surface area involvement). Allergic rhinitis severity was measured by using the Rhinitis Control Scoring System (RCSS) score (the maximum score is 50, corresponding to most severe case, and the minimum score is 10) (Table I). At this time, her RCSS score is 41. Skin prick, intradermal, and serum allergen–specific IgE testing results were positive for rough pigweed, white ash, red cedar, cedar elm pollen, shagbark hickory, sugar maple, dock-sorrel mix, lamb's quarter, common mugwort, English plantain, sheep sorrel, Bermuda grass, Johnson grass, and cat dander.

**Table I:** Cluster SCIT schedule

Phase	Visit	Dose No.	Dose volume	Dilution
Buildup	Visit 1	1	0.05 mL	1:1000
		2	0.10 mL	1:1000
		3	0.20 mL	1:1000
		4	0.30 mL	1:1000
		5	0.40 mL	1:1000
		6	0.50 mL	1:1000
	Visit 2	7	0.05 mL	1:100
		8	0.10 mL	1:100
		9	0.20 mL	1:100
		10	0.30 mL	1:100
		11	0.40 mL	1:100
		12	0.50 mL	1:100
	Visit 3	13	0.05 mL	1:10
		14	0.10 mL	1:10
		15	0.20 mL	1:10
	Visit 4	16	0.30 mL	1:10
		17	0.40 mL	1:10
		18	0.50 mL	1:10
	Visit 5	19	0.05 mL	1:1
		20	0.07 mL	1:1
	Visit 6	21	0.10 mL	1:1
	Visit 7	22	0.20 mL	1:1
	Visit 8	23	0.30 mL	1:1
	Visit 9	24	0.40 mL	1:1
Maintenance			0.40 mL	1:1

SCIT includes rough pigweed, white ash, red cedar, cedar elm pollen, shagbark hickory, sugar maple, dock-sorrel mix, lamb's quarter, common mugwort, English plantain, sheep sorrel, Bermuda grass, Johnson grass, and cat dander.

The patient’s atopy was unresponsive to standard-of-care first-line therapies, including levocetirizine, montelukast, topical hydrocortisone, and topical triamcinolone. She started taking subcutaneous dupilumab, 300 mg every 2 weeks, without any benefit.

Her treatment was then switched from dupilumab to upadacitinib, 15 mg daily, and cluster SCIT. Her SCIT schedule is summarized in Table I. The SCIT buildup phase was completed over the course of 1 month without any adverse effects. After maintenance dose SCIT had been achieved, the patient’s atopy remained in remission (RCSS score 10), even when upadacitinib was discontinued 5 months later. The patient continues to be followed 16 months after starting SCIT, with a maximum RCSS score after SCIT initiation of 18. She does not require any rescue medication for her atopic dermatitis or allergic rhinitis and conjunctivitis, and she continues to tolerate SCIT well without any adverse effects.

JAK inhibitors and anti-CD20 mAbs represent the growing adoption of medications targeting immunologic pathways. However, the effects of these medications on SCIT remain understudied. Although there is currently no globally accepted consensus on contraindications to SCIT, many allergy society guidelines suggest that immunosuppressive agents such as ofatumumab are contraindications for SCIT.^5^

In an Ask The Expert hosted by the American Academy of Allergy, Asthma & Immunology, ocrelizumab, another anti-CD20 mAb for MS, was hypothesized to make SCIT ineffective.^6^

Separately, a murine model of allergic asthma, showed that tofacitinib, another JAK inhibitor, in combination with SCIT suppressed lung eosinophil infiltration and local cytokine production better than SCIT alone, suggesting greater efficacy.^7^ However, to our knowledge, no clinical studies of JAK inhibitors and SCIT have been conducted.

Other mAbs, particularly omalizumab, have been used in conjunction with immunotherapy. A review of 5 double-blind, placebo-controlled trials and 3 observational studies demonstrated that omalizumab significantly reduced adverse events and decreased the time required to reach maintenance doses of SCIT.^8^ Furthermore, another randomized controlled trial of patients with house dust mite–driven asthma receiving either omalizumab alone, SCIT alone, or omalizumab and SCIT, showed a significant reduction in inhaled corticosteroid use in patients treated with omalizumab and SCIT versus in patients treated with omalizumab alone or SCIT alone.^9^

Here, we have demonstrated that anti-CD20 mAbs do not preclude a safe response to SCIT. A limitation of our work is that our patient was receiving SCIT and upadacitinib concurrently; thus, we are unable to ascertain whether clinical improvement was attributable to SCIT and/or upadacitinib. Similarly, it is not possible to disentangle the effects of anti-CD20 mAbs and JAK inhibitors on SCIT in our study. Additionally, because our patient remains in the maintenance phase of SCIT, the permanence of treatment response after SCIT cessation is unknown. Our findings should be confirmed in long-term prospective studies evaluating the drug classes separately.

Consent: We carefully explained to the subject the nature of this project and collected signed consent, and we hereby certify that to the best of our knowledge, the patient clearly understood what was involved in her participation and that her signature was legally valid. A medical problem or language or educational barrier did not preclude this understanding.

Data sharing statement: All relevant deidentified data and study materials are stored in a Health Insurance Portability and Accountability Act–compliant, password-protected, cloud-based storage. Access to these files will be provided on reasonable request to the corresponding author, John Leung.

## Disclosure statement

Disclosure of potential conflict of interest: J. Leung is a consultant for Devine, Millimet and Branch Professional Education, Sanofi, Huron Consulting Services LLC, Takeda, Ribon Therapeutics, Tegus, Slingsho, Guidepoint, Cowen, and AstraZeneca. Ther rest of the authors declare that they have no relevant conflicts of interest.
